# Supplementary material for: Sociodemographic determinants of multidrug-resistant tuberculosis in Lesotho: A case-control study
Source: PLOS Glob Public Health. 2025 Jul 10;5(7):e0004075. doi: 10.1371/journal.pgph.0004075 (PMC12244824; doi:10.1371/journal.pgph.0004075)
Supplement: Attachment — Submitted filename: Response to Reviewers.docx [file pgph.0004075.s004.docx]

37 Middlesex Circle Apt 10

Waltham, MA 02452

United States.

April 6, 2025

Editor-in-Chief
PLOS One Global Health

Dear Editor,

Thank you very much for your constructive feedback and the opportunity to revise and resubmit my manuscript entitled "Sociodemographic Determinants of Multidrug-Resistant Tuberculosis in Lesotho: A Case-Control Study" for publication consideration in your esteemed journal. I greatly appreciate the valuable insights and recommendations from both you and the reviewers. Below, I provide my detailed responses to each specific comment raised:

**Editor Comment:**
1. ***"The general introduction can be cut down and specific details on MDR-TB need to be added."***

**Response:**
I have revised the Introduction section by significantly condensing the background information and focusing explicitly on multidrug-resistant tuberculosis (MDR-TB). Specifically, I added detailed descriptions regarding MDR-TB prevalence, its impact on public health, current challenges associated with diagnosis and treatment, and the unique context of MDR-TB in Lesotho. These modifications enhance clarity and directly align the introduction with the primary objectives and findings of our study.

**Comment 2:**
"It will be useful to specify categorical variables with reference to age, and gender (male and female) should be shown separately. Odds ratios need to be presented separately along with the reference variable."

**Response:**
Thank you for this suggestion. I have clearly specified categorical variables for age groups (18–29, 30–39, 40–49, 50–59, and ≥60 years), with the youngest group (18–29 years) set as the reference. Gender is now explicitly separated as male and female, with male gender serving as the reference category. All odds ratios are presented separately and clearly indicate their respective reference groups in the revised Table.

**Comment 3:**
"The data has a significant number of women than men. Can authors explain the reasons for this gender difference? Are there more women than men affected with TB?"

**Response:**
I appreciate the opportunity to clarify this point. The predominance of female participants in the study reflects similar trends documented in previous surveys conducted in Lesotho, such as the 2014 Lesotho Demographic and Health Survey, which reported a higher TB burden among women. This may be attributed to sociocultural factors and gender roles, as women are more likely to seek healthcare due to their caregiving roles within families and communities, thus resulting in higher recorded TB incidence among women.

**Comment 4:**
"Discussion: Following sentence needs to be reworded. Instead of writing that income is a protective factor, authors can write that patients belonging to higher socioeconomic classes are less likely to get MDR-TB infection."

**Response:**
Thank you for the suggestion. I have rephrased this statement in the Discussion section to clearly state: "Patients belonging to higher socioeconomic classes were less likely to develop MDR-TB infection compared to those from lower socioeconomic classes."

**Comment 5:**
"The table on socio-demographic characteristics needs to be presented before the regression analysis."

**Response:**
Thank you for this recommendation. I have repositioned the socio-demographic characteristics table to appear before the regression analysis in the revised manuscript, ensuring logical flow and clarity.

**Reviewers Comments**

**Methodological Concerns:**

**Comment 6:**
"Sampling strategy: The manuscript does not provide detailed information on the sampling strategy used to select participants."

**Response:**
I have expanded the Sampling Strategy section in the manuscript to include detailed information regarding the proportionate stratified random sampling approach. Specifically, I clarified the method used to randomly select participants within each clinic, ensuring proportional representation and generalizability of the findings.

**Comment 7:**
"Data collection methods: The study relies on phone surveys and face-to-face interviews, which may introduce differences in data quality."

**Response:**
While I acknowledge this concern; I have however, clarified that the survey instrument was standardized for all participants. Prior to data collection, the questionnaire was rigorously tested among the target population and demonstrated consistent understanding and ease of response. Consequently, there were no observed differences in the quality or nature of responses between phone surveys and face-to-face interviews.

**Comment 8:**
"Variable definitions: Some variables, such as caregiver support, are not clearly defined, which may lead to ambiguity in interpretation."

**Response:**
Thank you for highlighting this issue. I have clarified the definition of caregiver support in the Methods section. Specifically, I defined caregiver support as "the presence of a designated family member, friend, or community health worker who assists the participant regularly in managing medication adherence, clinic visits, and overall TB treatment compliance."

**Statistical Concerns:**

**Comment 9:**
"Model specification: The logistic regression model does not account for potential interactions between variables."

**Response:**
Thank you for raising this point. I have re-evaluated the logistic regression model and explored potential interactions among key sociodemographic variables, such as between age and income, and gender and caregiver support. However, these interaction terms were not statistically significant and were thus excluded from the final model to maintain parsimony and interpretability.

**Comment 10:**
"Model fit: The manuscript does not report on the model's goodness-of-fit statistics."

**Response:**
I have addressed this concern by including the Hosmer–Lemeshow goodness-of-fit statistic (χ² = 6.24, p = 0.6205), which indicates adequate model fit. In addition, I reported the model's pseudo R² value (0.04) and information criteria (AIC = 397.37, BIC = 438.07) to provide a comprehensive understanding of the model's performance.

**Comment 11:**
"Variable selection: The study does not provide a clear rationale for the selection of variables included in the logistic regression model."

**Response:**
I appreciate this observation. In the revised manuscript, we now specify that variables were selected based on both theoretical relevance as identified in the literature and empirical associations observed in bivariate analyses with a p-value threshold of <0.25. This approach ensured that potentially meaningful variables were retained in the multivariate analysis while controlling for confounding.

I believe these revisions have strengthened our manuscript and improved clarity. We look forward to your consideration of the revised manuscript and are happy to address any further points if required.

Sincerely,

Jerry Yakubu Yahaya (PhD)
Ghana AIDS Commission
jerrywayo123@gmail.com
