# Supplementary material for: Sociodemographic determinants of multidrug-resistant tuberculosis in Lesotho: A case-control study
Source: PLOS Glob Public Health. 2025 Jul 10;5(7):e0004075. doi: 10.1371/journal.pgph.0004075 (PMC12244824; doi:10.1371/journal.pgph.0004075)
Supplement: Attachment — Submitted filename: Response_to_Reviewers_auresp_2.docx [file pgph.0004075.s005.docx]

37 Middlesex Circle Apt 10

Waltham, MA 02452

United States.

May 27, 2025.

Editor-in-Chief
PLOS One Global Health

Dear Editor,

Thank you once again for your detailed feedback on my manuscript titled Sociodemographic Determinants of Multidrug-Resistant Tuberculosis in Lesotho: A Case-Control Study”. I appreciate the opportunity to revise my submission and respond to the helpful suggestions made by both the editor and reviewers. Below is my point-by-point response:

### **Reviewer Comment 1:**

**Line 4 –** "This study aimed to identify key sociodemographic determinants associated with MDR-TB among adult TB patients in Lesotho." This sentence in the abstract is repeated.

**Response:**
The repeated sentence has been removed to avoid redundancy and improve the clarity of the abstract.

### **Reviewer Comment 2:**

***The “Ethics statement” should be placed at the beginning of the Methods section.***

**Response:**
As suggested, I have moved the “Ethics statement” to the beginning of the Methods section to align with the journal’s formatting and structural requirements.

### **Editorial Comment:**

***Please review your reference list to ensure that it is complete and correct. If you have cited papers that have been retracted, please include the rationale for doing so in the manuscript text, or remove these references and replace them with relevant current references. Any changes to the reference list should be mentioned in the rebuttal letter.***

**Response:**
I have reviewed and revised the reference list thoroughly to ensure accuracy, completeness, and relevance. Below is a summary of the changes made:

#### **A. References Removed from the Final Manuscript:**

As advised in the initial editorial review, a significant portion of the manuscript especially within the **Introduction and Discussion** sections were condensed or removed to comply with editorial recommendations for clarity. As a result, some references that were originally cited to provide contextual background, methodological precedent, or regional comparison were no longer explicitly referenced in the revised version. These include:

1. Centers for Disease Control and Prevention. (2021). Transmission and spread of tuberculosis.
2. World Health Organization. (2018). Tuberculosis factsheet.
3. Brooks-Pollock E, et al. (2011). Epidemiologic modeling for TB control in South Africa. PNAS.
4. World Health Organization. (2018). Tuberculosis Q&A.

Additionally, the following older or less directly relevant references were removed and replaced with more recent sources:

6. Moyo S, et al. (2016). MDR-TB outcomes in adolescents, South Africa. PLoS ONE.
7. Moreno-Serra R, et al. (2022). Recall bias in conflict zones. Conflict and Health.
8. Schwarzer R. (2014). Health behavior: Theory, research, and practice.
9. Brisbin S, et al. (2020). Alcohol/tobacco and TB outcomes. J Subst Abuse Treat.

#### **B. Updated and Current References Included:**

The following **new references** were added to enhance the study’s empirical grounding, provide updated evidence, and broaden the geographical and conceptual relevance of the findings. These additions fall into five key thematic categories:

#### **1. Global and Regional MDR-TB Trends and Risk Factors**

These references were added to position Lesotho's MDR-TB burden within a global context and draw parallels with similar high-burden countries:

- **Xi et al. (2022):** A recent systematic review and meta-analysis offering a comprehensive assessment of global MDR-TB risk factors.
- **Song et al. (2024):** Presents a 30-year global trend analysis of MDR-TB, strengthening the epidemiological context of the study.
- **Cameron et al. (2020), Zhang et al. (2019), Baya et al. (2019), Afshari et al. (2019), Ali et al. (2019), Senanayake et al. (2018), Stosic et al. (2018):** These regional case-control and cross-sectional studies from Zimbabwe, China, Mali, Iran, Sudan, Sri Lanka, and Serbia respectively, provide comparable evidence from diverse settings that align with the study findings on age, income, and sociodemographic determinants of MDR-TB.

#### **2. Lesotho-Specific and Sub-Saharan Africa Context**

These sources were added to enrich the country-specific focus and reflect the most current national and subregional data:

- **WHO African Region – Lesotho Country Disease Outlook (2023):** Provides updated statistics and strategic outlook on TB in Lesotho.
- **Lesotho Ministry of Health (2020);** National Tuberculosis and Leprosy Control Programme Annual Report 2020. Informed the study design and background, though the citation was not maintained in the final narrative.
- **Hirsch-Moverman et al. (2022):** Highlights the effectiveness of community-based interventions in Lesotho.
- **Luba et al. (2019):** Offers key demographic insights from a population-based study in Lesotho that supported the baseline data analysis of the study.

#### **3. Social Determinants and Economic Impacts**

To support the manuscript’s emphasis on income and caregiver support as predictors, the following references were introduced:

- **Duarte et al. (2018), Wingfield et al. (2018), Fuady et al. (2018):** These works emphasize the importance of social determinants and financial barriers in TB outcomes and supported the findings regarding poverty and caregiver roles.
- **Lopez et al. (2019)**: Provided a behavioral psychology perspective on treatment adherence, reinforcing the value of caregiver support.
- **Brito et al. (2020):** A meta-analysis underscoring how social support significantly influences TB treatment success.

#### **4. HIV Co-Infection and Controversies in the Literature**

To present a balanced view on the role of HIV in MDR-TB, the following current and diverse studies were included:

- **Suthar et al. (2012):** Shows the protective role of antiretroviral therapy (ART) in TB prevention.
- **Sultana et al. (2021):** A meta-analysis that supports an association between HIV and MDR-TB risk.
- **Wilson et al. (2020), Mishkin et al. (2018), Gandhi et al. (2013)**: Present contrasting findings regarding HIV as a risk factor in both low- and high-resource settings.

#### **5. TB Prevention and Behavioral Risk Factors**

These references offered insight into behavioral dimensions relevant to TB transmission and progression, which were incorporated into the covariate discussion:

- **Johnson et al. (2018):** Focuses on TB burden among older adults and supported our age-related findings.
- **Senanayake et al. (2018)** and **Sharma et al. (2019):** Provided evidence on social and lifestyle factors associated with TB/MDR-TB in developing countries.

These references were selected for their rigor, relevance, and contribution to the themes of the manuscript. I trust these additions have improved the overall quality, depth, and scientific integrity of the manuscript.

Sincerely,
Dr. Jerry Yahaya
